# The Efficacy of Transversus Abdominis Plane (TAP) Blocks When Completed by Anesthesiologists Versus by Surgeons: A Systematic Review and Meta-Analysis

**DOI:** 10.3390/healthcare12242586

**Published:** 2024-12-22

**Authors:** Dylan Irvine, Christopher Rennie, Emily Coughlin, Imani Thornton, Rahul Mhaskar, Jeffrey Huang

**Affiliations:** 1 HCA Florida Westside Hospital, Plantation, FL 33324, USA; 2 Dr. Kiran C. Patel College of Osteopathic Medicine, Nova Southeastern University, Clearwater, FL 33759, USA; cr2338@mynsu.nova.edu; 3 Morsani College of Medicine, University of South Florida, Tampa, FL 33602, USA; 4 Department of Anesthesiology and Critical Care, Moffitt Cancer Center, Tampa, FL 33612, USA

**Keywords:** transversus abdominis plane blocks, TAP blocks, ultrasound-guided, laparoscopic-guided, perioperative medicine, anesthesiology, regional anesthesiology

## Abstract

**Background/Objectives**: Current literature has demonstrated the benefits of transversus abdominis plane (TAP) blocks for reducing postoperative pain and opioid consumption for an array of surgical procedures. Some randomized controlled trials and retrospective studies have compared ultrasound guidance TAP blocks completed by anesthesiologists (US-TAP) to laparoscopic guidance TAP blocks completed by surgeons (LAP-TAP). However, the findings of these studies have not been consolidated to improve recommendations and patient outcomes. Our objective is to consolidate and summarize current literature regarding the efficacy of TAP blocks for postoperative pain control and opioid consumption when performed with ultrasound guidance (US-TAP, compared to laparoscopic guidance (LAP-TAP). **Methods**: We performed a systematic review and meta-analysis of RCTs and retrospective studies to evaluate US-TAP versus LAP-TAP blocks for postoperative pain control and opioid consumption. We searched PubMed/MEDLINE, CINAHL, Cochrane, and Web of Science databases for all articles meeting the search criteria until the time of article extraction in February 2024. The primary outcome variables were postoperative pain scores and opioid consumption. The secondary outcome variables were complications, time taken to perform the block, length of stay (LOS) in the hospital, and cost of performing the block. **Results**: Of the 1673 articles initially identified, 18 studies met the inclusion criteria for evaluation. Of the included studies, 88.9% and 77.8% found no significant difference in postoperative pain scores or opioid consumption, respectively, between US-TAP and LAP-TAP groups. Six studies (33.3%) found that LAP-TAP was faster to perform than US-TAP. Meta-analysis demonstrated no statistically significant differences in postoperative pain scores or opioid consumption between groups but showed that block times were significantly longer in the US-TAP group. **Conclusions**: US-TAP and LAP-TAP blocks may be equivocal in terms of reducing postoperative pain and opioid consumption. LAP-TAPs may be less time-consuming and more cost-effective and viable alternatives to US-TAP blocks in the perioperative setting.

## 1. Introduction

Transversus abdominis plane (TAP) blocks involve administration of local anesthetic into two distinct layers of muscle, known as the fascial plane, between the transversus abdominis (TA) and the internal oblique (IO) muscles. Similarly, subcostal TAP blocks are performed in the fascial plane found between the TA and the rectus abdominis (RA). This technique effectively provides analgesia for surgical procedures involving the abdomen via nerve blockade of the anterior rami of T6 to L1 [1]. TAP blocks are a commonly used form of ultrasound-guided somatic analgesia in the setting of abdominal surgery. The use of a TAP block was first documented in 2001, with increasing utilization over the last two decades as ultrasound technology has continued to advance [2,3]. High-quality studies have also compared TAP blocks to wound infiltration (WI) with local anesthetic and have shown that TAP blocks provide better postoperative pain control than local WI, significantly lowering pain scores at multiple time points [4]. Additionally, TAP blocks reduce opioid consumption compared to WI, which may help improve recovery and decrease opioid-related side effects [4].

As the use of TAP blocks increases, indications for this procedure are further expanding. Most major procedures involving surgical exploration of the abdominal cavity can benefit from the administration of a TAP block [2,5,6]. TAP blocks have been shown to lower postoperative pain scores, decrease postoperative opioid consumption, decrease the need for anti-emetics, reduce opioid-related complications, and improve patient satisfaction regarding pain management [7,8]. TAP blocks can be a critical component of postoperative pain management, and they play a crucial role in Enhanced Recovery After Surgery (ERAS) protocols, as they significantly affect recovery and patient well-being. Proper pain control helps reduce the need for opioids, supports early mobilization, speeds up gastrointestinal recovery, and shortens hospital stays, all of which contribute to a smoother recovery process and a better overall patient experience.

Despite these advantages, one major concern associated with TAP blocks is the inability to provide visceral pain management [2,9]. Furthermore, there are concerns about rare, albeit possible, complications associated with TAP blocks, such as femoral nerve palsy, local hematoma formation, bowel perforation, and intra-abdominal organ damage such as hepatic, splenic, and renal lacerations [5,10]. TAP blocks now traditionally utilize ultrasound guidance instead of relying solely on landmark palpation for placement, and thus, the rates of these complications have decreased significantly [11].

Some notable differences between ultrasound- versus laparoscopic-guided TAP blocks exist. Ultrasound assistance involves identifying the transversus abdominis plane using real-time imaging, inserting a needle through the wall of the abdomen to inject anesthetic into the plane [12]. In laparoscopic blocks, insufflation of CO_2_ into the abdomen during abdominal procedures is one approach that can provide space to aid in identifying the abdominal wall layers and facilitates the injection of anesthetic directly into the appropriate plane [12]. Laparoscopic blocks require larger doses of anesthetic in the abdominal wall compared to US-guided blocks to achieve adequate block duration and analgesia, due to more precise targeting of the transversus abdominis plane. However, the volume of anesthetic injected into the plane will vary depending on the type and formulation of local anesthetic used.

There is debate within anesthesia and surgical communities to about when a TAP block should be performed and by whom. Anesthesiologists commonly administer TAP blocks via ultrasound assistance (US-TAP), but they can also be performed intraoperatively by surgeons through laparoscopic assistance (LAP-TAP) [13,14]. Some may prefer surgeon-placed TAP blocks for their cost-effectiveness and convenience. However, there remains a current gap in the literature surrounding the comparative risks and benefits of TAP blocks, whether they are performed by an anesthesiologist or a surgeon. Additionally, there are limited generalizable data across various surgical interventions to support recommendations for either type of TAP block. Here, we aim to synthesize the current literature available regarding the comparative efficacy of TAP blocks performed by anesthesiologists and surgeons. The primary aims of this review are to assess postoperative pain control and opioid consumption.

## 2. Materials and Methods

For the purposes of this review, we sourced studies regarding transversus abdominis plane (TAP) blocks placed by anesthesia providers versus surgeons to assess their efficacy for controlling postoperative pain, opioid consumption, and the incidence of complications or adverse effects in the perioperative period according to the Preferred Reporting Items for Systematic Reviews and Meta-Analyses guidelines. The study protocol was registered in the International Prospective Register of Systematic Reviews (PROSPERO) (ID# CRD42024520718).

Two investigators performed this search in the following databases: PubMed/MEDLINE, CINAHL, the Cochrane Library, and the Web of Science.

The search terms used in this literature review were as follows: “transverse abdominis plane block” OR “transversus abdominis plane block” OR “TAP block” OR “Nerve Block/methods” OR “quadratus lumborum block” OR “erector spinae block” AND “surgeon” OR “laparoscopic” OR “laproscop” AND “anesthesiolog” OR “ultrasound-guided” OR “ultrasound”.

This literature search was performed in February 2024. Only studies published or available in English were considered in this systematic review. Inclusion criteria limited these articles to randomized controlled studies and observational studies that focused on postoperative opioid consumption, pain scores, or incidence of complications or adverse effects of TAP blocks when performed by anesthesia providers or surgeons for patients in the perioperative period. Studies were not limited by the timeframe of publication, patient age, or type of surgery. We included all studies in which TAP blocks were included as part of the perioperative analgesia plan and where anesthesiologist- versus surgeon-administered TAP blocks were compared within the same study. We scanned the citations of included studies in relevant articles and references, cross-referencing them with similar systematic reviews to ensure comprehensive indexing. We focused solely on peer-reviewed published studies and excluded gray literature. Additionally, we applied filters for systematic review keywords and publication types to exclude systematic review articles.

The results obtained from the original database search were subsequently screened manually by the two investigators for the stated inclusion criteria. If studies met all relevant inclusion criteria but lacked substantial pertinent data, they were excluded from the review. After initial exclusion by two independent researchers, full texts of the studies were read and evaluated. Relevant data were extracted from the selected studies if they continued to meet inclusion criteria. Both researchers assessed the study quality independently, and a consensus was reached on the articles to be included in the analysis. If required, a third team member reviewed the discrepancy until a consensus was reached. All reviews were performed blind, and any discrepancies regarding inclusion were later discussed and agreed upon. The Cochrane risk-of-bias tool for randomized trials was used to score all the articles before inclusion. We used the ROBINS-1 tool for non-randomized studies to evaluate observational studies included in this review. Only studies with a perceived “low risk of bias” (Cochrane risk-of-bias tool) or “low risk of bias judgement” (ROBINS-1) were included in this systematic review. Only studies with low risk of bias were included to ensure the results are accurate and trustworthy. Including studies with high or unclear risk of bias can introduce systematic errors, which may distort the overall findings and limit the generalizability of the conclusions. Further, we did not include observational studies in the meta-analysis to prevent their data from biasing the results.

The screened articles were recorded in Table 1, which contains the following columns: study title, year of publication, study design, patient population inclusion criteria, clinical outcomes evaluated, and main results. Only articles deemed eligible for inclusion by concurrent agreement were subsequently analyzed. The primary outcome variables for analysis were postoperative opioid consumption and postoperative pain scores. Secondary outcome variables for analysis included complications or adverse effects from the block, hospital length of stay, cost of performing block, and time taken to perform the block.

Data were extracted as means and standard deviations for the continuous outcomes of pain score, opioid requirement, and block time. In cases where the median and range were reported, these were converted to the mean and standard deviation using methods described by Hozo et al. [15]. Data for these outcomes were pooled using a random effects model, and pooled mean difference (MD) and 95% confidence intervals (CIs) were reported. Heterogeneity was assessed using the I2 statistic, with >50% considered moderate heterogeneity and >80% considered significant heterogeneity. Subgroup analyses were used to assess different measurement scales and, when possible, separate studies with a pediatric population. Analysis was completed using Review Manager software version 5.4.

**Table 1:** Summary of data from included studies [16,17,18,19,20,21,22,23,24,25,26,27,28,29,30,31,32,33].

Study	Year	Study Design	Patients	Outcomes Evaluated	Main Results
Civitella et al. [16]	2023	RCT	*N* = 60, radical prostatectomy, US-TAP (*n* = 30) or LAP-TAP (*n* = 30).	Post-op pain scores, opioid consumption, complications, LOS.	No significant difference in post-op pain scores, opioid consumption, complications, or LOS between groups.
Diyaolu et al. [17]	2021	RCT	*N* = 50, pediatric laparoscopic procedures, US-TAP (*n* = 25) or LAP-TAP (*n* = 25).	Post-op pain scores, opioid consumption, block completion time.	No significant difference in post-op pain scores or opioid consumption between groups. Blocks were faster in the LAP-TAP group compared to the US-TAP group (2.1 ± 1.9 min vs. 7.9 ± 3.4 min, *p* < 0.001).
Doble et al. [18]	2018	Retrospective review	*N* = 39, hernia repair, US-TAP (*n* = 17) or LAP-TAP (*n* = 22).	Post-op pain scores, opioid consumption, LOS.	Post-op pain score (2.35 vs. 4.18, *p* = 0.019) and opioid consumption during hospitalization (408.52 vs. 860.92 mg; *p* = 0.013) were lower in the LAP-TAP group compared to the US-TAP group. No significant difference in LOS between groups.
Emile et al. [19]	2022	RCT	*N* = 110, laparoscopic cholecystectomy, US-TAP (*n* = 36), LAP-TAP (*n* = 36), or no TAP (*n* = 38).	Post-op pain scores, opioid consumption, complications.	US-TAP and LAP-TAP groups had lower pain scores and opioid consumption compared to the no TAP group. No significant differences between US-TAP and LAP-TAP groups. No complications reported in any group.
La Regina et al. [20]	2023	RCT	*N* = 112, colorectal surgery, US-TAP (*n* = 57) or LAP-TAP (*n* = 55).	Post-op pain scores, opioid consumption, complications, LOS.	No significant difference in post-op pain scores, opioid consumption, complications, or LOS between groups.
Landmann et al. [21]	2018	Retrospective review	*N* = 330, pediatric cases, US-TAP (*n* = 125), LAP-TAP (*n* = 88), or local wound infiltration (*n* = 117).	Post-op pain scores, opioid consumption, LOS.	No significant difference in post-op pain scores or opioid consumption between groups. LAP-TAP group demonstrated a shorter LOS compared to the other groups (*p* = 0.02).
McDonald et al. [22]	2022	RCT	*N* = 78, gynecology oncology cases, US-TAP (*n* = 39), or LAP-TAP (*n* = 39).	Post-op pain scores, opioid consumption.	No difference in pain scores between groups. Significant increase in opioid consumption among LAP-TAP group compared to US-TAP group in first 24 hr post-op (*p* = 0.018).
Narasimhulu et al. [23]	2018	RCT	*N* = 41, cesarean sections, US-TAP (*n* = 21), or LAP-TAP (*n* = 20).	Post-op pain scores, opioid consumption, block completion time.	No significant difference in post-op pain scores or opioid consumption between groups. Block completion time was significantly less for the LAP-TAP group compared to the US-TAP group (2.4 vs. 12.1 min, *p* <0.001).
Paasch et al. [24]	2020	Retrospective review	*N* = 116, inguinal hernia repair, US-TAP (*n* = 58) or LAP-TAP (*n* = 58).	Post-op pain scores, opioid consumption.	No significant difference in post-op pain scores or opioid consumption between groups.
Park et al. [25]	2017	RCT	*N* = 80, laparoscopic colectomy, US-TAP (*n* = 40), or LAP-TAP (*n* = 40).	Post-op pain scores, opioid consumption.	No significant difference in post-op pain scores or opioid consumption between groups.
Ravichandran et al. [26]	2017	RCT	*N* = 60, laparoscopic cholecystectomy, US-TAP (*n* = 30), or LAP-TAP (*n* = 30).	Post-op pain scores, opioid consumption, block completion time.	No significant difference in post-op pain scores or opioid consumption between groups. Block completion time was significantly less for the LAP-TAP group compared to the US-TAP group (*p* < 0.05).
Sahap et al. [27]	2023	RCT	*N* = 63, laparoscopic cholecystectomy, US-TAP (*n* = 21), LAP-TAP (*n* = 21), or no TAP (*n* = 21).	Post-op pain scores, opioid consumption.	No significant difference in post-op pain scores or opioid consumption between the US-TAP and LAP-TAP groups. Pain scores were significantly lower in the US-TAP and LAP-TAP groups compared to the no TAP group (*p* < 0.05).
Sharma et al. [28]	2023	RCT	*N* = 122, bariatric surgery, US-TAP (*n* = 60), or LAP-TAP (*n* = 62).	Post-op pain scores, opioid consumption, block completion time, cost of performing block.	No significant difference in post-op pain scores or opioid consumption between groups. LAP-TAP had shorter block completion time compared to US-TAP (3.58 min vs. 12.47 min, *p* < 0.01). The cost of US-TAP was USD 50 greater, on average, than that of LAP-TAP.
Soyturk el al. [29]	2023	RCT	*N* = 170, laparoscopic cholecystectomy, US-TAP (*n* = 55), LAP-TAP (*n* = 59), or no TAP (*n* = 56).	Post-op pain scores, opioid consumption.	The US-TAP and LAP-TAP groups had lower pain scores and opioid consumption compared to the no TAP group. Pain scores 1 and 12 h post-op were higher in the US-TAP group compared to the LAP-TAP group (*p* < 0.001). No significant differences in opioid consumption between US-TAP and LAP-TAP groups.
Urfalioglu et al. [30]	2017	RCT	*N* = 75, cesarean section, US-TAP (*n* = 38), or LAP-TAP (*n* = 37).	Post-op pain scores, opioid consumption, complications, block completion time.	No significant difference in post-op pain scores, opioid consumption, or complications between groups. Average block completion time was longer in the US-TAP group than in the LAP-TAP group (10 min vs. 7 min).
Venkatraman et al. [31]	2020	RCT	*N* = 80, laparoscopic cholecystectomy, US-TAP (*n* = 40) or LAP-TAP (*n* = 40).	Post-op pain scores, opioid consumption.	No significant difference in post-op pain scores between groups. Post-op opioid consumption was less in the US-TAP group than in the LAP-TAP group (*p* = 0.049).
Wong et al. [32]	2020	RCT	*N* = 60, laparoscopic colectomies, US-TAP (*n* = 31), or LAP-TAP (*n* = 29).	Post-op pain scores, opioid consumption, complications.	No significant difference in post-op pain scores, opioid consumption, or complications between groups.
Zaghiyan et al. [33]	2019	RCT	*N* = 107, laparoscopic colorectal surgery, US-TAP (*n* = 45), LAP-TAP (*n* = 41), or no TAP (*n* = 21).	Post-op pain scores, opioid consumption, complications, LOS.	No significant difference in post-op pain scores, complications, or LOS between all groups. The LAP-TAP group had less post-op opioid consumption compared to both the US-TAP (*p* = 0.007) and no TAP (*p* = 0.007) groups.

Legend. RCT: randomized controlled trial; N: sample size; US-TAP: ultrasound-guided transversus abdominis plane block; LAP-TAP: laparoscopic-guided transversus abdominis plane block; LOS: length of stay; Min: minutes; Hr: hour.

## 3. Results

### 3.1. Included Studies

**Figure 1:**
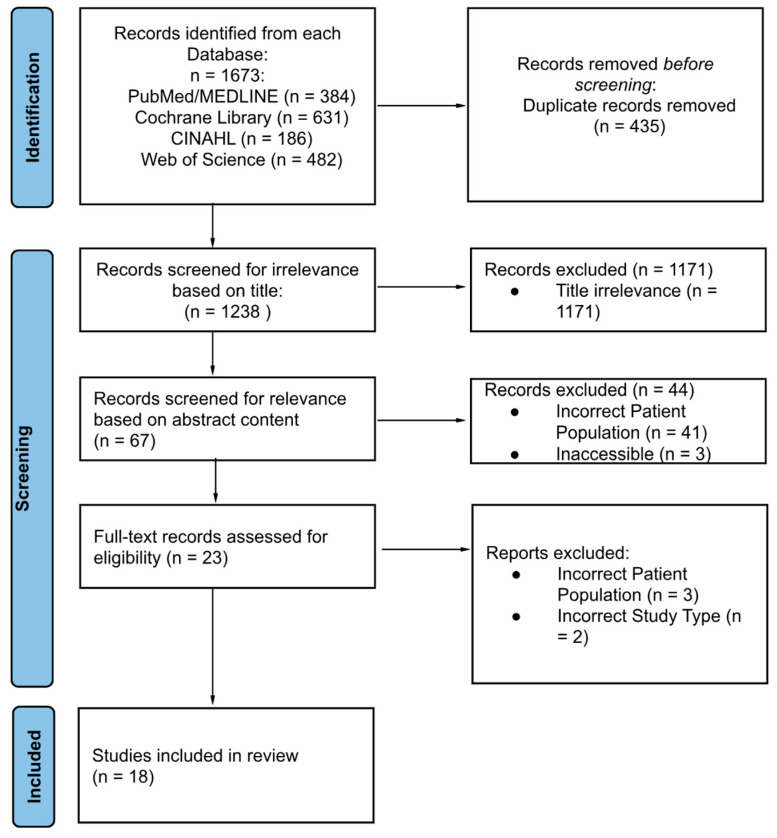
PRISMA flow chart—identification included studies from a search of databases.

As seen in Figure 1, 1673 articles were identified in the primary search across all databases. Following a review to assess eligibility based on our inclusion and exclusion criteria, 18 publications were deemed eligible for subsequent qualitative analysis.

### 3.2. Main Results

As outlined by our inclusion criteria, the eighteen included studies were all published or available in English [16,17,18,19,20,21,22,23,24,25,26,27,28,29,30,31,32,33]. Of the eighteen included studies, fifteen (*n* = 15, 83.3%) were randomized controlled trials (RCTs) and three (*n* = 3, 16.7%) were retrospective studies. A total of 1753 patients were included across all studies. The number of participants included in each study ranged from 39 to 330, with the average being 97.4 patients [16,17,18,19,20,21,22,23,24,25,26,27,28,29,30,31,32,33]. Patient demographic data such as age, sex, gender and race were not unanimously reported for each patient in every publication, thus are not presented here. Table 1 depicts the data from each included study, with information about the authorship, study design, patients, clinical outcomes evaluated, and main results.

Of the included studies, two studies (*n* = 2, 11.1%) involved pediatric patients undergoing various undisclosed surgical cases where TAP blocks were being performed [17,21]. The remaining studies each identified a single type of surgical case among the included patients: laparoscopic cholecystectomy (*n* = 5, 27.7%) [19,26,27,29,31], colorectal surgery (*n* = 4, 22.2%) [20,25,32,33], cesarean section (*n* = 2, 11.1%) [23,30], hernia repair (*n* = 2, 11.1%) [18,24], prostatectomy (*n* = 1, 5.6%) [16], gynecologic oncology (*n* = 1, 5.6%) [22], and bariatric surgery (*n* = 1, 5.6%) [28].

Of the included studies, thirteen (*n* = 13, 72.2%) involved two arms and compared ultrasound-guided TAP (US-TAP) blocks placed by anesthesiologists to laparoscopic-guided TAP blocks placed by surgeons (LAP-TAP) [16,17,18,20,22,23,24,25,26,28,30,31,32]. One study (*n* = 1, 5.6%) involved three arms and compared US-TAP, LAP-TAP, and a control group who received local wound infiltration which was performed by a surgeon intraoperatively [21]. Four studies (*n* = 4, 22.2%) involved three arms and compared US-TAP, LAP-TAP, and a group of controls who received no TAP block or other form of perioperative regional anesthesia [19,27,29,33]. Of the included patients within these studies, 43.8% (*n* = 768) received an ultrasound-guided TAP block from an anesthesiologist (US-TAP), 41.7% (*n* = 732) received a laparoscopic-guided TAP block from a surgeon (LAP-TAP), 6.7% (*n* = 117) were controls who received local wound infiltration by a surgeon, and 7.8% (*n* = 136) were controls who received no TAP block.

### 3.3. Effect on Pain Scores

Of the included studies, all eighteen (*n* = 18, 100.0%) reported postoperative pain scores between US-TAP and LAP-TAP groups. Sixteen studies (*n* = 16, 88.9%) found no difference in postoperative pain scores between US-TAP and LAP-TAP groups [16,17,19,20,21,22,23,24,25,26,27,28,30,31,32,33]. The remaining two studies (*n* = 2, 11.1%) found reduced postoperative pain scores in the LAP-TAP group compared to the US-TAP group [18,29].

**Figure 2:**
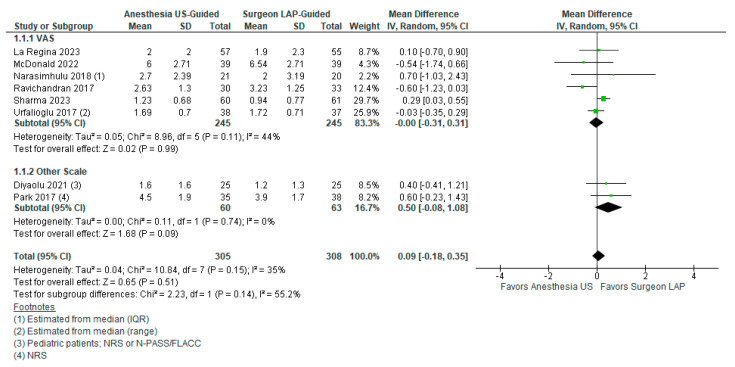
Postoperative pain scores between US-TAP and LAP-TAP groups [17,20,22,23,25,26,28,30].

Of the included studies, eight (*n* = 8, 44.4%) studies provided data for pain scores from either VAS or NRS scales and were included for meta-analysis. These studies included 305 patients in the US-TAP and 308 in the LAP-TAP groups. There was no significant difference between groups for pain score (MD 0.09, 95% CI −0.18–0.35). Subgroups assessing VAS and NRS/other scales separately showed that studies utilizing VAS scales favored US-TAP while studies utilizing NRS or other scales favored LAP-TAP. However, both subgroups showed no significant differences. The overall heterogeneity among studies for this outcome was low at I2 = 35%, *p* = 0.15. These findings are displayed in Figure 2.

### 3.4. Effect on Postoperative Opioid Consumption

Of the included studies, all eighteen (*n* = 18, 100.0%) reported on postoperative opioid consumption between US-TAP and LAP-TAP groups. Fourteen studies (*n* = 14, 77.8%) found there was no difference in postoperative opioid consumption between the US-TAP and LAP-TAP groups [16,17,19,20,21,23,24,25,26,27,28,29,30,32]. Two studies (*n* = 2, 11.1%) found reduced postoperative opioid consumption in the US-TAP groups [22,31]. Two studies (*n* = 2, 11.1%) found reduced postoperative opioid consumption in the LAP-TAP groups compared to the US-TAP groups [18,33].

**Figure 3:**
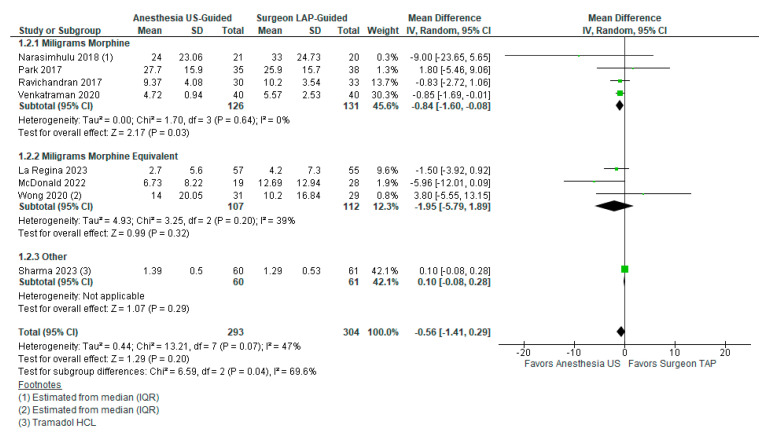
Postoperative opioid consumption between US-TAP and LAP-TAP groups [20,22,23,25,26,28,31,32].

Of the included studies, eight (*n* = 8, 44.4%) studies included quantitative data for opioid requirements and were included for meta-analysis. A total of 293 patients were in the US-TAP group and 304 were in the LAP-TAP group. There was no significant difference in opioid requirement between groups (MD −0.56, 95% CI −1.41–0.29). Three (*n* = 3, 16.7%) studies reported on the number of milligrams of morphine administered, which revealed lower opioid requirements in the US-TAP group (MD −0.84, 95% CI −1.60–(−0.08)). Three (*n* = 3, 16.7%) studies reported the milligrams of morphine equivalent; these studies showed a lower opioid requirement among the US-TAP group, but this difference was insignificant (MD −1.95, 95% CI −5.79–1.89). One (*n* = 1, 5.6%) study specifically reported only use of Tramadol HCL, which showed no difference. The heterogeneity among studies for this outcome was moderate at I2 = 47%, *p* = 0.07. These findings are displayed in Figure 3.

### 3.5. Block Completion Time

Of the included studies, five (*n* = 5, 27.8%) reported the duration required to perform the TAP block between the US-TAP and LAP-TAP groups. All five studies (*n* = 5, 27.8%) found that block completion time was significantly longer in the US-TAP groups compared to the LAP-TAP groups [17,23,26,28,30].

**Figure 4:**
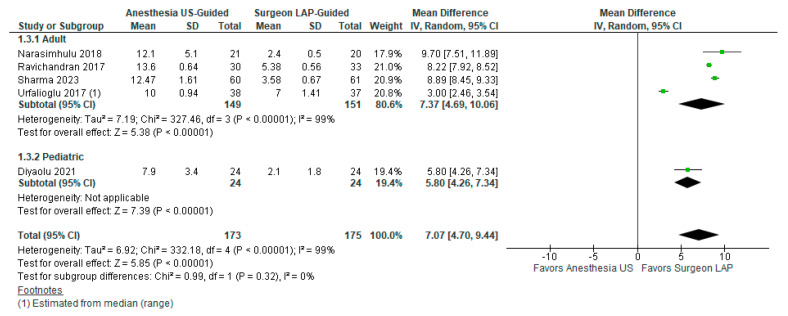
Block completion time between US-TAP and LAP-TAP groups [17,23,26,28,30].

Of the five (*n* = 5, 27.8%) studies, 173 patients were in the US-TAP group and 175 patients were in the LAP-TAP group. Block time was significantly higher within the US-TAP group (MD 7.07, 95% CI 4.70–9.44). There was significant heterogeneity among studies reporting block time (I2 = 99%, *p* < 0.0001). These findings are displayed in Figure 4.

### 3.6. Incidence of Complications/Adverse Effects

Of the included studies, six (*n* = 6, 33.3%) reported the incidence of complications between the US-TAP and LAP-TAP groups. All six studies (*n* = 6, 33.3%) found no significant difference in the incidence of complications between the US-TAP and LAP-TAP groups [16,19,20,30,32,33].

### 3.7. Hospital Length of Stay (LOS)

Of the included studies five (*n* = 5, 27.8%) reported the hospital length of stay (LOS) between the US-TAP and LAP-TAP groups [16,18,20,21,33]. Four studies (*n* = 4, 22.2%) found no significant difference in LOS in the hospital between the US-TAP and LAP-TAP groups [16,18,20,33]. One study (*n* = 1, 5.6%) found that the LOS in the hospital was shorter in the LAP-TAP group compared to the US-TAP group [21].

### 3.8. Cost of Performing Block

Of the included studies, one (*n* = 1, 5.5%) reported the cost of performing TAP blocks between US-TAP and LAP-TAP groups. This study found that US-TAP was more expensive than LAP-TAP, incurring costs of an additional USD 50 per block on average [28].

## 4. Discussion

This systematic review aimed to provide a qualitative analysis of the current literature regarding ultrasound-guided TAP blocks placed by anesthesiologists compared to laparoscopic-assisted TAP blocks placed by surgeons. The results of our search and extensive sorting through articles for our inclusion criteria limited our review to 18 eligible studies [16,17,18,19,20,21,22,23,24,25,26,27,28,29,30,31,32,33].

The primary outcome variables for analysis in our review were postoperative pain scores and opioid consumption. Pain scores are subjective and rely on individual patient reporting, which can introduce variability. However, pain scores remain the most widely accepted and practical tool for assessing pain in clinical studies. Although objective measures such as physiological markers or advanced imaging techniques have been explored, they have not yet been established as reliable standalone indicators of pain, nor are they routinely available in clinical settings. Pain is inherently a subjective experience, influenced by psychological, cultural, and individual factors, making patient-reported outcomes like pain scores the closest representation of the patient’s experience. Many high-quality studies continue to use validated pain scales (e.g., the Visual Analog Scale or the Numeric Rating Scale) as a cornerstone for evaluating pain management strategies. These tools are validated, simple to administer, and allow for comparisons across diverse patient populations and interventions. While we recognize the limitations of pain scores, they remain essential for gauging the efficacy of interventions.

Our review demonstrates that approximately 88.9% and 77.8% of the included studies found no significant differences in postoperative pain scores or opioid consumption, respectively, between US-TAP and LAP-TAP groups. Of the remaining studies, findings between groups were mixed, with approximately half finding improved outcomes in the US-TAP groups and the other half in the LAP-TAP groups. Our study corroborates other literature that has demonstrated improved postoperative pain control and reduced opioid consumption in patients undergoing eligible surgeries who receive a TAP block in the perioperative period, compared to controls [34,35,36]. However, this review suggests that there are marginal differences in these primary outcomes irrespective of whether the TAP block is performed by an anesthesiologist or a surgeon.

This can likely be attributed to the fact that both US-TAP and LAP-TAP blocks involve injection of local anesthetic into the same plane, one block via ultrasound guidance and the other with laparoscopy. These difference approaches both contribute to effective analgesia in the same plane.

Whether an anesthesiologist performs an intraoperative LAP-TAP or a preoperative US-TAP is performed by the surgeon, the literature supports our finding that there is minimal difference in postoperative pain and analgesic load. Notably, several studies in recent years have found that postoperative administration may provide the most clinically applicable benefits with regard to pain and opioid consumption [37,38]. Additionally, it is difficult to extrapolate differences in postoperative pain scores and narcotic necessity due to several circumstantial factors. One study suggests that while TAP blocks are effective, there are large variations in efficacy depending on the type and location of the abdominal surgery, citing potential benefits of subcostal blocks for upper-abdominal procedures [7]. As this review included seven different forms of abdominal surgery in both pediatric and adult patients, it was unsurprising to find variable results between the efficacy of US-TAP and LAP-TAP. Beyond this, numerous patient factors play a role in the multifaceted experience of pain. Preoperative pain sensitivity, pain threshold, mental health disorders such as anxiety and depression, and prior trauma all play a major role in the perception of pain, which is difficult to control in a comparison such as this [39,40].

The secondary outcome variables used for analysis in our review included complications or adverse effects from the block, block completion time, LOS in the hospital, and the cost of performing the block. Our review demonstrated that among the included studies, 27.8% of studies reported on the time taken to perform the block and unanimously reported that LAP-TAP was faster to perform than US-TAP [17,23,26,28,30]. Further, US-TAP was reported as more expensive to perform than LAP-TAP, adding an additional cost to the perioperative period [28]. The association between quicker block times and reduced cost among LAP-TAP blocks is likely due to the reduced turnaround time, as well as factors such as the elimination of additional skin prep, ultrasound machine setup, and team switching that are offered by the LAP-TAP approach compared to US-TAP. There were no significant differences in complications between groups [16,19,20,30,32,33]. When used in conjunction with minimally invasive surgery, TAP blocks play a key role in enhancing postoperative recovery by providing pain relief, reducing opioid use, and promoting early mobilization. These benefits may lead to shorter hospital stays and quicker recovery times, which could improve patient outcomes and reduce healthcare costs. Although these data would be difficult to collect and quantify, in a macroeconomic context, the widespread adoption of TAP blocks could decrease the need for extended pain management and reduce the overall financial burden on healthcare systems, making it a cost-effective approach to patient care.

Ultimately, the findings of our review emphasize that LAP-TAP placed by surgeons may be an equivocal alternative to the more conventional US-TAP placed by anesthesiologists for benefits with postoperative pain and opioid consumption, with the added benefits of lowering block time and cost and without an increased risk of complications.

The current literature supports these findings, with studies detailing the advantages of LAP-TAP, such as lower rates of visceral injury and incorrect plane blockade [41]. These benefits provide a large cost benefit in the context of factors such as complication management and length of stay. Another primary advantage of LAP-TAP with regard to block time and cost is the ability to achieve direct visualization [41,42]. Laparoscopic camera assistance presents a small time burden, as the equipment is typically already established intraoperatively. Additionally, studies have shown that LAP-TAP cases may offer potential cost-effectiveness and may be suitable for meeting inhaled anesthetic agent requirements [43].

The authors wish to illustrate the numerous strengths of this study. This study provides, to our knowledge, the first systematic review and meta-analysis that compares the postoperative pain scores, opioid consumption, and other outcome variables between US-guided TAP blocks placed by anesthesiologists and LAP-assisted TAP blocks placed by surgeons. Specifically, our review encompasses 18 studies with a total of 1753 patients, grouped by similar surgical procedures where applicable. We acknowledge that including diverse surgical procedures introduces heterogeneity; however, it was necessary to achieve adequate statistical power given the current scarcity of direct comparisons. Further, this literature review used a robust, broad search sequence which was applied to multiple databases to comprehensively evaluate the current literature. Blinded reviews performed by two independent reviewers allowed the articles to be sorted and agreed upon in a non-biased way. The data presented here allow us to report with confidence that the value of TAP blocks for reducing postoperative pain and opioid consumption is likely not affected by whether the block is US-guided by an anesthesiologist or LAP-assisted by a surgeon.

Although our study has inherent strengths, there are some limitations to address. There are still limited RCTs available that compare TAP blocks placed by anesthesiologists to those placed by surgeons. The 18 studies included in this review had a mean sample size of 97.4 patients [16,17,18,19,20,21,22,23,24,25,26,27,28,29,30,31,32,33]. Including some studies with small sample sizes makes it difficult to draw significance from the data reported. For example, our sample size (*n* = 348) and the significant heterogeneity in our meta-analysis regarding block completion time limits the significance of this finding. Larger sample sizes would likely reduce the heterogeneity of our findings and strengthen our ability to make recommendations to guide clinical decisions regarding US- versus LAP-assisted TAP blocks. Further, multiple studies used different measures for outcome variables, such as postoperative pain scores and opioid consumption. For example, some studies used inconsistent pain scales, follow-up intervals, and analgesic medications, which reduced the number of studies that could be included in the meta-analysis. Not all studies included in our manuscript contained data regarding all of the outcome variables we aimed to assess. Also, our specific search strategy may have omitted potentially relevant studies from our search, and searching more databases for articles to assess may have increased the amount of studies and data available for analysis.

Future studies should strive to compare postoperative pain scores and opioid requirements in a large sample of patients and report on these outcomes using standardized measures such as the VAS and oral morphine equivalents at consistent follow-up intervals. In addition, encouraging future studies to include standardized reporting of pain scores at defined intervals postoperatively will be essential. We hope that our review will encourage both surgeons and anesthesiologists to engage in more rigorous and comparative research, thereby strengthening the evidence base in this area.

## 5. Conclusions

TAP blocks have been proven to be an effective component of the anesthesia care plan for reducing postoperative pain and opioid consumption for patients undergoing a variety of surgical procedures. However, it is unclear if there are significant benefits in patient outcomes when these blocks are administered through ultrasound guidance by anesthesiologists versus through laparoscopic guidance by surgeons. The decision regarding who should perform the TAP block is determined by the perioperative care team, and could be aided by future studies that compare the outcomes emphasized in this manuscript using a large sample of patients and standardized measures for pain scales and opioid consumption. Both anesthesia providers and surgeons are qualified to administer the block, enhancing postoperative analgesia for patients.
